# Cyanoacrylate versus suture as flap closure methods in mandibular third molar surgery: a split-mouth randomized controlled clinical study

**DOI:** 10.4317/medoral.26375

**Published:** 2024-06-22

**Authors:** Margalida Santmartí-Oliver, Santiago Bazal-Bonelli, Luis Sánchez-Labrador, Tomás Beca-Campoy, Fabián Pérez-González, Carlos Manuel Cobo-Vázquez, Cristina Madrigal Martínez-Pereda, Cristina Meniz-García

**Affiliations:** 1 Department of Dental Clinical Specialties, Faculty of Dentistry, Complutense University of Madrid, Spain; 2 Department of Surgery, Biomedical Research Institute, University of Salamanca, Spain

## Abstract

**Background:**

Sutures have been the standard flap closure method of choice following mandibular third molar surgery but can lead to some complications. Tissue adhesives, including cyanoacrylate, have emerged as alternative flap closure method in this surgery to overcome such drawbacks. However, limited clinical trials can be found. Therefore, the aim of this clinical study was to compare two methods of flap closure in mandibular third molar surgery, cyanoacrylate and 4/0 silk sutures, by assessing post-operative outcome measures (pain, swelling, trismus, and healing) and patient-reported outcome measures (PROMs).

**Material and Methods:**

A randomized split-mouth clinical trial was designed, in which mandibular third molar (M3M) extractions were performed, where the control side flap was closed with 4/0 silk sutures and the test side flap with cyanoacrylate. Swelling, pain, trismus, healing, and PROMs were recorded post-operatively. These variables were analyzed by means of the nonparametric Mann-Whitney U test, using SPSS statistical software version 28.0.0 (IBM® SPSS®, Chicago, IL, USA). For all results, a 95% confidence interval was recorded (significance level *p* < 0.05, two-tailed).

**Results:**

A total of 17 patients were recruited and 34 mandibular third molar extractions were performed. No statistically significant differences were found in terms of swelling, pain, trismus, healing, and PROMs between both groups (*p*<0.05).

**Conclusions:**

No statistically significant differences were found between flap closure with 4/0 silk sutures and cyanoacrylate, in terms of surgical post-operative outcomes and PROMs. However, further studies with larger sample sizes are required to be able to affirm it with greater certainty.

** Key words:**Tooth extraction, suture, cyanoacrylate, third molar.

## Introduction

Mandibular third molar (M3M) surgery is the most frequent surgical procedure performed by oral and maxillofacial surgeons. Its most prevalent post-operative complications include pain, swelling, trismus and, to a lesser extent, alveolar osteitis (1).

Sutures have been the standard flap closure method of choice following M3M surgery, to achieve primary closure and ensure effective hemostasis. Sutures also allow for tissue repositioning to either their original or alternative positions, control alveolar bone exudate and prevent dislodgment of the blood clot from the extraction socket (2).

However, sutures may also act as a foreign body and promote microbial colonization and adhesion, in turn leading to an inflammatory response (3). In addition, inadequate force control during tissue manipulation and needle penetration can lead to tears, wound dehiscence, or flap ischaemia. These complications can in turn cause infectious complications and/or flap necrosis, which impede healing and affect the post-operative period. These complications linked to sutures have led to the search for alternative flap closure methods in M3M surgery. Tissue adhesives have gained popularity in the literature as a potential method to overcome such drawbacks (4).

As of today, cyanoacrylate is the most widely used tissue adhesive. Its excellent tensile strength, rapid polymerization, biocompatibility, immediate hemostasis, ease of application, bacteriostatic properties and improved healing make it an attractive option for oral surgical procedures. It is non-resorbable and takes 7 to 10 days to detach from the oral mucosa (5).

However, there are a limited number of clinical trials (6-8) that assess post-operative surgical outcomes such as pain, swelling, trismus and healing when comparing flap closure with cyanoacrylate and sutures in M3M surgery. To our knowledge, no clinical trial has assessed, to date, patient-reported outcome measures (PROMs) between the two methods. Therefore, the aim of this study was to assess and compare cyanoacrylate and 4/0 silk sutures as flap closure methods in M3M surgery, in terms of post-operative outcome measures (pain, inflammation, trismus and healing) and PROMs.

## Material and Methods

- Study design

This study was designed as a split-mouth randomized prospective clinical trial in compliance with the Helsinki Declaration for research involving human subjects. The study protocol was approved by the Ethics Committee of the San Carlos Clinical Hospital in Madrid, registration code 20/711-EC_X, dated December 22, 2020.

- Participants

Patients who attended the Oral Surgery and Implant Dentistry Program at the Faculty of Dentistry of Complutense University of Madrid for the bilateral extraction of M3Ms were selected and enrolled. Selection criteria were as follows:

a) Inclusion criteria:

1. 18 years of age.

2. Indication for bilateral extraction of M3Ms, with the most symmetrical pre-operative surgical difficulty according to Pederson's difficulty scale (9).

3. No active periodontal disease.

4. No allergies to any components of the cyanoacrylate employed for wound closure in the study (Epiglu®, Meyer-Haake GmbH Medical Innovations, Germany).

b) Exclusion criteria

1. Refusal to participate in the study after having been informed of the study characteristics and requirements for participation.

2. Inability to attend follow-up visits 48 hours and 1 week post-operatively.

3. Smoking 10 cigarettes/day.

4. Medical comorbidities including immunosuppression or impaired tissue healing (including diabetes type I or II), or bleeding disorders.

5. Undergoing active antibiotic or anticoagulant therapy, and/or having taken anti-inflammatory drugs within 4 days prior to the procedure.

6. Need for antibiotic prophylaxis prior to surgery.

7. Pregnancy or breastfeeding.

- Interventions

**Figure 1:**
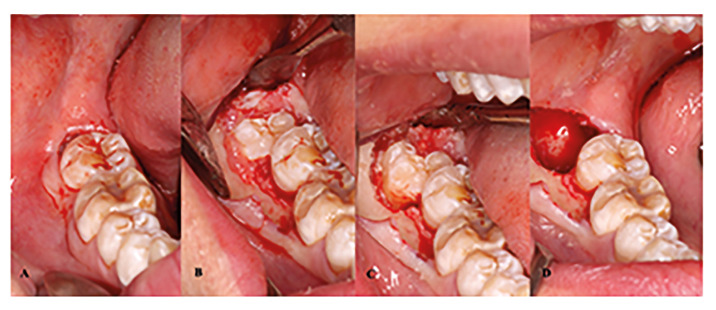
Surgical procedure. A: Baseline; B: Mucoperiosteal flap reflection; C: Bone removal; D: Extraction socket.

All M3M extractions were standardized and performed by the same clinician (S.B.B). The local anaesthetic agent of choice was 4% articaine with 1:100,000 adrenaline (Ultracaín, Normon SL, Madrid, Spain) to anaesthetize the inferior alveolar, lingual, and long buccal nerves. A full-thickness mucoperiosteal envelope flap was raised in all cases. When necessary, bone removal and/or tooth sectioning were performed with a cooled sterile saline-irrigated surgical handpiece and a tungsten carbide round bur. The M3Ms were extracted with the use of elevators. Sharp bony socket edges were smoothened, followed by curettage of the distal aspect of the second molar, and socket lavage with sterile saline solution (Fig. 1).

**Figure 2:**
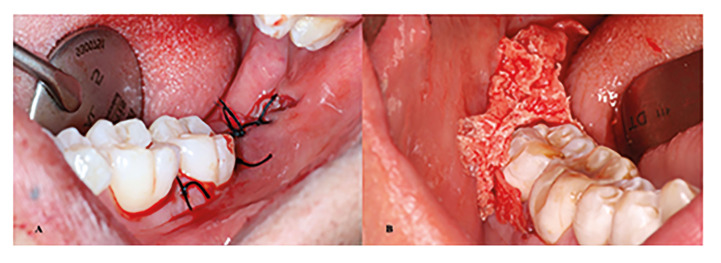
Flap closure. A: Suture; B: Cyanoacrylate.

Flap closure was performed with 4/0 silk sutures in the control group (Aragó, Barcelona, España) and cyanoacrylate in the test group (Epiglu, Meyer-Haake GmbH Medical Innovations, Germany), allocated according to prior randomization (Fig. 2). Suture placement varied based on flap extension: either one or two simple interrupted sutures in the distal aspect of the second molar, a Figure of eight suture or a horizontal mattress suture in the distal relieving incision, and a simple interrupted in the mesial papillae of the second molar. Compression for 30 minutes with a sterile gauze pack was indicated.

With regards to flap closure in the test group with cyanoacrylate, tissue forceps were used to pull upward from the most distal portion of the distal relieving incision, aiming to approximate and tighten the wound margins. Once the wound edges were securely in place, a layer of cyanoacrylate was spread along the incision margins up to the mesial aspect of the mandibular second molar, ensuring proper coverage and sealing. The surgery was concluded after waiting 30 to 60 seconds to achieve proper polymerization, following the manufacturer's instructions.

Patients were provided with written and verbal post-operative instructions and prescribed the following medication: amoxicillin 750 mg every 8 hours for 7 days, ibuprofen 600 mg every 8 hours for 5 days, and paracetamol 650 mg every 8 hours as a rescue analgesic.

Post-operative examinations were performed 48 hours and 7 days after surgery (when suture or cyanoacrylate were removed). Extraction of the contralateral M3M was planned for one month after the first extraction, following the same standardized surgical plan, but flap closure being achieved with the unperformed method.

- Outcomes

Pre-operative outcomes (secondary variables):

Patient characteristics (demographic data and medical history): age, sex, general health status, comorbidities, medication, and tobacco and alcohol consumption.

M3M characteristics: indication for extraction (caries, pericoronitis, others), position (partially erupted, submucosal or impacted) or angulation (vertical, horizontal, mesioangular, distoangular, or inverted).

Intra-operative outcomes (secondary variables):

Surgical time: from the beginning of the incision to flap closure or application of cyanoacrylate (in minutes).

Surgical difficulty according to Parant's scale (10).

Post-operative outcomes (primary variables):

Post-operative pain: patients were instructed to record perceived pain levels using a Visual Analog Scale (VAS) at 6 hours after surgery and at 9 PM for 7 consecutive days, with the endpoints being "no pain" (0) and "extreme pain" (10). The number of rescue analgesics (1, 2 or 3 per day) during 7 days after surgery were also recorded.

Swelling: assessed by measuring facial perimeter using anatomical reference points: distance in millimeters from tragus to the labial commissure (Trg-com), from tragus to pogonion (Trg-Pg), and from the lateral corner of the eye to the gonial angle (Go-eye), following the modified criteria proposed by Amin and Laskin (11). Measurements were taken before surgery, at 48 hours, and 7 days after surgery.

Trismus: measured by assessing maximum mouth opening capacity using a manual caliper (interincisal distance or ID; expressed in mm) before surgery, at 48 hours, and 7 days after surgery.

Wound healing: assessed using a qualitative scale based on a previous study (12) at 48 hours and 7 days after surgery:

1. Good: aesthetic, clean and good opposing wound edges; colour of the mucosa identical to the surrounding area; no dehiscence.

2. AccepTable: slightly irregular wound edges, light bleeding or erythema; colour of the mucosa similar to the surrounding area, 1-2mm dehiscence.

3. Bad: irregular wound edges, moderate or heavy bleeding, exudate, pus, foul odor, signs of infection; erythematous oral mucosa; dehiscence >2mm, open wound, keloid formation or unaesthetic closure.

**Figure 3:**
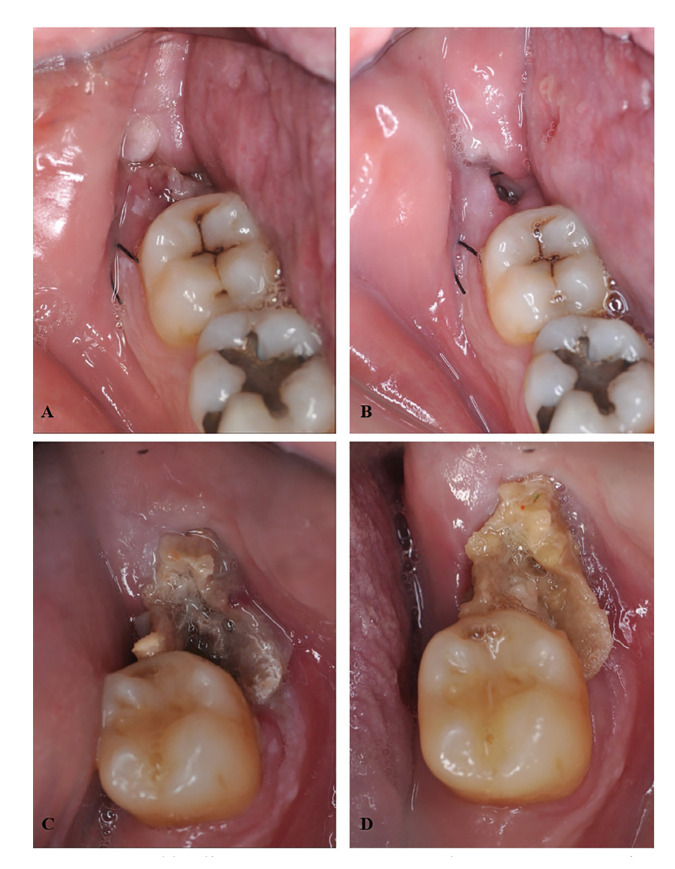
Wound healing assessment. A: Control group, post-operative day 2; B: Control group, post-operative day 7; C: Test group, post-operative day 2; D: Test group, post-operative day 7.

Clinical photographs were also taken for further assessment (Fig. 3).

PROMs: to assess PROMs according to the flap closure method used, the Oral Health Impact Profile (OHIP-14) survey (13) was conducted on post-operative day 7.

- Sample size

Sample size calculation was performed with G Power 3.1 software (Dusseldorf, Germany) based on the pain data according to the Visual Analog Scale (VAS) on post-operative day 1 described by Oladega *et al*. (8) in their study. An effect size of 1.5 was yielded, which, combined with an α error of 0.05 and an 80% statistical power, resulted in a total sample of 14 patients. However, considering the 15% expected dropout rate in dental clinical trials (14), a minimum of 16 patients were deemed necessary for this study, following a split-mouth design.

- Randomization and blinding

Randomisation was restrictive or balanced, as all patients received treatment for flap closure. Investigator bias in case allocation was prevented with the use of Viedoc® software (Pharma Consulting Group, Uppsala, Sweden) by the principal investigator (C.M.G), uninvolved in the selection, treatment, and evaluation of patients or in the analysis of results and did not have access to patients' pre-operative characteristics, characteristics of the M3Ms, as well as the surgical procedure’s characteristics.

The operator (S.B.B) was only informed about the intervention group, i.e., the flap closure method, during the final phase of the surgery. Blinding was also implemented so that the evaluator and person responsible for measurements (L.S.-L.) was not present during randomization or surgery. Therefore, whilst the evaluator knew the intervention group (due to the impossibility of blinding the flap closure method), he also was unaware of the patients' pre-operative characteristics and those of the M3Ms, as well as the details of the surgical procedure. It was impossible to achieve blinding of the study subjects due to the different nature of the flap closure methods used in this study, in which patients underwent local anesthesia for surgical procedures.

- Statistical methods

The collected data were entered into an Excel spreadsheet (MS Excel 2019, Microsoft Inc., Redmond, WA, USA) and sent to an independent statistician for further analysis using SPSS statistical software, version 28.0.0 (IBM® SPSS®, Chicago, IL, USA).

Descriptive statistics were firstly obtained for all variables (frequency, mean, standard deviation, median, minimum, and maximum), and the normality of the sample was assessed with the Shapiro-Wilk test. The differences between the study variables of the test and control groups were determined. The variables "indication for extraction," "third molar position," "third molar angulation," "surgical difficulty," and "wound healing" were analyzed using the chi-square test, whilst the variables "VAS pain score," "number of rescue analgesics", "swelling", “trismus", "patient satisfaction level", and "surgical time" were analyzed using the non-parametric Mann-Whitney U test, as the variables did not follow a normal distribution. For all results, a 95% confidence interval was recorded (significance level *p* < 0.05, two-tailed).

## Results

- Pre and intra-operative outcomes

**Figure 4:**
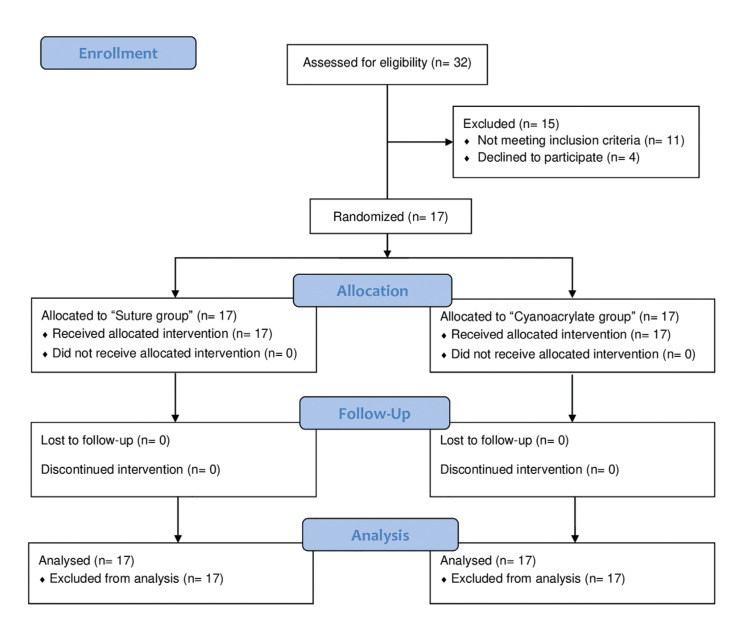
CONSORT participant flow-diagram.

A total of 17 patients were included in this study, 8 males and 9 females, with an average age of 26.65 ± 8.20 years. No patients were lost or excluded during follow-up. A flow-chart of patient participation in this study is shown in Fig. 4. Given the split-mouth design of the study, the total sample consisted of 34 M3M (17 in both test and control groups respectively). No statistically significant differences (*p*=1.000) were found between both groups in terms of M3M characteristics. Information about indication for extraction, M3M position and angulation, and surgical difficulty and duration are shown in Table 1 and Table 2. There were no statistically significant differences (*p*=1.000) between the test and control groups in relation to these variables.

- Post-operative outcomes

Post-operative pain reached its peak score at 6 hours post-operatively for both the control and test groups, with a score of 6.47 ± 2.035 and 6.82 ± 1.468 (*p*=0.790) respectively. No statistically significant differences were found in any of the post-operative pain outcome measures. With regards to the need for rescue analgesia, the highest intake was also observed on the same day of the procedure in both groups. No statistically significant differences were found between each group.

The maximum swelling values, measured from different facial perimeter measurements (Go - eye, Trg - com, and Trg - Pg), were found on post-operative day 2 in both groups, with no statistically significant differences.

No noTable differences were seen in maximum mouth opening values between both groups; no statistically significant differences were found between the groups.

Pain, swelling and trismus data are summarised in Table 3.

On post-operative day 2, wound healing in the test group was accepTable in all cases except one, where it was deemed to be bad (94.1% and 5.9%, respectively). In the control group, two cases (11.8%) displayed good healing, whilst 15 cases (88.2%) showed accepTable healing.

On post-operative day 7, seven cases (41.2%) in both groups showed good healing and 10 cases (58.8%) demonstrated accepTable healing. No statistically significant differences were found between the group (*p*>0.05).

In reference to PROMs, it is noteworthy that the average total scores were very similar for both groups. In both control and test groups, the lowest scores were seen in the domain of physical pain, which included questions regarding tooth sensitivity and dental pain. However, no statistically significant differences were found between any of the domains or total scores (Table 4)

## Discussion

M3M surgery is invariably related with post-operative complications, including pain, swelling and trismus (1). Therefore, numerous studies (15-17) have been carried out in an attempt to improve the post-operative outcomes of this procedure, including the method of flap closure, which remains a challenge (6-8,18-21). Traditionally, sutures have been used for this purpose, but due to their numerous drawbacks (3), tissue adhesives such as cyanoacrylate have begun to be used as a suggested alternative.

In the present randomized clinical trial, 34 M3Ms were extracted bilaterally in 17 patients in whom no statistically significant differences were found in terms of pain, swelling, trismus, soft tissue healing and PROMs, in contrast to the latest systematic review (22), which concluded that cyanoacrylate showed better results in terms of pain, bleeding and swelling when compared to flap closure with sutures.

Although the surgical time was slightly longer in the test group, but not statistically significant (*p*=0.761), as shown in Table 2, there were no statistically significant differences compared to the control group in terms of pain, swelling, trismus and healing. Possible explanations for the increased surgical time could be the surgeon's lack of experience in handling this product, the lack of specific guidelines for the intraoral use of cyanoacrylate, its rapid polymerization, or the need to open a second unit due to insufficient quantity for adequate closure. However, in general, shorter time needed for flap closure decreases the total tissue manipulation time, reducing surgical trauma and, therefore, postoperative complications (23).

Some authors (7,18) have observed a statistically significant reduction in post-operative pain scores assessed by VAS when tissue adhesives are used versus sutures. Gogulanathan *et al*. (18) described that this reduction could be explained by the sealing effect of the fibrin adhesive on the exposed nerve endings. However, neither Ghoreishian *et al*. (6) nor Oladega *et al*. (8) found statistically significant differences in pain scores between the two groups, consistent with the results found in this randomized clinical trial, neither in terms of post-operative pain scores nor intake of rescue analgesics.

In this clinical trial, in line with the findings by Oladega *et al*. (8), no statistically significant differences were found between the two study groups in terms of post-operative swelling. On the other hand, Kulkarni *et al*. (24) found in their study that there was significantly less post-operative swelling in sites in which cyanoacrylate had been used for flap closure instead of sutures, explaining that the difference could be due to suture-triggered foreign body reaction and greater plaque accumulation on the suture threads. However, the lack of differences in surgical time and difficulty between both groups in this study could explain the lack of differences in swelling, since as reported by Cobo-Vazquez *et al*. (25), prolonged surgical time in M3M surgery is associated with increased swelling.

With regards to wound healing, in this study, no significant differences were found between the study groups, in keeping with the results found by other authors, such as Oladega *et al*. (8), who assessed the wound dehiscence and surgical site infection separately.

To our knowledge, this study is the first to assess PROMs comparing two different methods of flap closure for M3M surgery. For both methods of wound closure, overall patient satisfaction was practically similar, and the domain with the worst result was physical pain, followed by psychological disability. However, on the other hand, there are numerous studies pertaining to different medical specialties, including traumatology (26), plastic surgery (27) or abdominal surgery (28), who have observed greater patient-reported satisfaction outcomes with cyanoacrylate when compared to conventional sutures. Further studies may hence be required to assess patient-reported satisfaction in the field of M3M surgery, in order to establish with greater certainty if there are significant changes in patient satisfaction.

Despite no statistically significant differences were found in the trial, possibly due to a small sample size, other studies (6-8) position cyanoacrylate similarly or even endorse it as superior to sutures for M3M extraction wound closure. These studies cite advantages like hemostasis, reduced pain and inflammation, lower skill demand, patient comfort by avoiding a second suture appointment, and quick, easy and effective application. However, tissue adhesives face limitations in high-tension areas, modified wound closure needs, infection zones, and large dead spaces. Drawbacks include product cost and potential allergies (4,29).

Regarding the limitations of this study, analysis of post-operative bleeding would also be of interest as it can influence the variables assessed in the current investigation, and confirm the reported trends related to cyanoacrylate in terms of improved hemostasis. Furthermore, higher operator experience with cyanoacrylate may lead to improved results of the analysed post-operative parameters, as cyanoacrylate is less commonly used in the field of oral surgery compared to sutures.

## Conclusions

The absence of statistically significant differences in the variables of this study (pain, swelling, trismus, healing and PROMs) suggest that whilst cyanoacrylate is not superior to sutures, it could be considered as effective as sutures in flap closure in M3M surgery. Whilst sutures are considered as the gold standard due to their economical price, ease in use and widespread use by oral and maxillofacial surgeons, further studies are required to establish a more definitive conclusion

## Figures and Tables

**Table 1:** Pre-operative and intra-operative characteristics for each patient.

Patient	Control group	Test group
Patient 1	Mesioangular	Mesioangular
	Partial coverage	Partial coverage
	Type II	Type II
Patient 2	Mesioangular	Mesioangular
	Partial coverage	Partial coverage
	Type II	Type II
Patient 3	Horizontal	Horizontal
	Partial coverage	Partial coverage
	Type III	Type III
Patient 4	Vertical	Horizontal
	Partial coverage	Partial coverage
	Type I	Type II
Patient 5	Mesioangular	Mesioangular
	Total coverage (submucosal)	Total coverage (submucosal)
	Type III	Type III
Patient 6	Vertical	Vertical
	Partial coverage	Partial coverage
	Type I	Type I
Patient 7	Mesioangular	Mesioangular
	Total coverage (submucosal)	Partial coverage
	Type III	Type III
Patient 8	Mesioangular	Mesioangular
	Partial coverage	Partial coverage
	Type II	Type II
Patient 9	Mesioangular	Mesioangular
	Partial coverage	Partial coverage
	Type III	Type III
Patient 10	Horizontal	Horizontal
	Total coverage (submucosal)	Total coverage (submucosal)
	Type III	Type III
Patient 11	Horizontal	Horizontal
	Total coverage (submucosal)	Total coverage (submucosal)
	Type III	Type III
Patient 12	Mesioangular	Mesioangular
	Total coverage (submucosal)	Total coverage (submucosal)
	Type II	Type II
Patient 13	Mesioangular	Mesioangular
	Total coverage (submucosal)	Total coverage (submucosal)
	Type II	Type II
Patient 14	Horizontal	Horizontal
	Partial coverage	Partial coverage
	Type III	Type II
Patient 15	Mesioangular	Mesioangular
	Total coverage (submucosal)	Total coverage (submucosal)
	Type III	Type III
Patient 16	Distoangular	Distoangular
	Partial coverage	Partial coverage
	Type II	Type II
Patient 17	Horizontal	Horizontal
	Total coverage (submucosal)	Total coverage (submucosal)
	Type II	Type II

**Table 2:** M3M Characteristics and intra-operative variables.

Characteristics		Control group (n=17)	Test group (n=17)	*p value*	Total (n=34)
Indication for extraction	Pain	13/17 (76.5%)	12/17 (70.6%)	1*	25/34 (73.5%)
	Tooth decay	2/17 (11.8%)	2/17 (11.8%)		4/34 (11.8%)
	Pericoronitis	2/17 (11.8%)	3/17 (17.6%)		5/34 (14.7%)
M3M angulation	Mesioangular	9/17 (52.9%)	9/17 (52.9%)	1*	18/34 (52.9%)
	Horizontal	5/17 (29.4%)	6/17 (35.3%)		11/34 (32.4%)
	Vertical	2/17 (11.8%)	1/17 (5.9%)		3/34 (8.8%)
	Distoangular	1/17 (5.9%)	1/17 (5.9%)		2/34 (5.2%)
M3M position	Partial coverage	9/17 (52.9%)	10/17 (58.8%)	1*	19/34 (55.9%)
	Total coverage (submucosal)	8/17 (47.1%)	7/17 (41.2%)		15/34 (44.1%)
	Total coverage (included)	0/17 (0%)	0/17 (0%)		0/34 (0%)
Surgical difficulty (14)	Type I	2/17 (11.8%)	1/17 (5.9%)	0.787*	3/34 (8.8%)
	Type II	7/17 (41.2%)	9/17 (52.9%)		16/34 (47.1%)
	Type III	8/17 (47.1%)	7/17 (41.2%)		15/34 (44.1%)
Mean intervention duration (minutes)		16.06	16.88	0.980†	16.47

*Chi - square test. †Mann-Whitney Test.

**Table 3:** Post-operative effects results.

Post-operative effects		Control group (n=17)		Test group (n=17)		p* value
		Mean	SD	Mean	SD	
Pain	6h post - procedure	6.47	2.035	6.82	1.468	0.790
	Day 2	5.76	2.513	5.59	2.599	0.850
	Day 3	4.82	2.038	5.06	2.193	0.672
	Day 4	3.47	1.736	3.71	1.795	0.701
	Day 5	2.06	1.345	2.35	1.579	0.660
	Day 6	1.06	1.197	1.35	1.320	0.520
	Day 7	0.71	0.920	0.88	0.993	0.647
Rescue analgesic consumption	6h post - procedure	1.29	0.686	1.41	0.507	0.775
	Day 2	0.88	0.781	0.94	0.748	0.878
	Day 3	0.29	0.470	0.41	0.507	0.721
	Day 4	0.12	0.332	0.12	0.332	1
	Day 5	0	0	0.06	0.243	1
	Day 6	0	0	0	0	1
	Day 7	0	0	0	0	1
Swelling	Go- eye: Baseline	106.06	1.784	107.47	2.065	0.030
	Go- eye: Day 2	115.88	4.270	116.06	4.763	0.726
	Go- eye: Day 7	110.06	2.680	110.94	2.839	0.326
	Trg-com.: Baseline	112.00	2.372	111.24	2.223	0.265
	Trg-com.: Day 2	116.47	2.478	116.00	3.062	0.502
	Trg-com.: Day 7	113.59	2.293	113.59	2.152	0.857
	Trg- Pg: Baseline	146.00	4.555	146.18	4.348	0.726
	Trg- Pg: Day 2	154.53	5.821	153.82	5.582	0.477
	Trg- Pg: Day 7	149.71	5.241	149.00	4.287	0.625
Trismus	Baseline	53.06	1.676	53.18	1.741	0.833
	Day 2	42.47	5.269	42.47	5.014	0.980
	Day 7	47.29	3.514	46.76	2.862	0.713

* Mann-Whitney Test.

**Table 4:** PROMs using the OHIP-14 questionnaire.

PROMs		Control group (n=17)		Test group (n=17)		p* value
Domain	Item	Mean	SD	Mean	SD	
Functional limitation	1	1.00	0	1.12	0.485	1
	2	1.00	0	1.06	0.243	1
Physical pain	3	2.41	0.939	2.35	0.931	0.991
	4	1.65	0.996	1.94	0.827	0.190
Psychological discomfort	5	1.06	0.243	1.06	0.243	1
	6	1.53	0.717	1.71	0.849	0.645
Physical disability	7	1.06	0.243	1.06	0.243	1
	8	1.00	0	1.00	0	1
Psychological disability	9	1.35	0.493	1.41	0.712	1
	10	1.59	1.004	1.65	0.862	0.722
Social disability	11	1,35	0,493	1.41	0.712	1
	12	1.06	0.243	1.06	0.243	1
Handicap	13	1.00	0	1.00	0	1
	14	1.00	0	1.00	0	1
Total score		18.06	1.919	18.82	2.921	0.321

* Mann-Whitney Test.
